# Placebo in Dental Practice

**Published:** 1873-07

**Authors:** J. C. Henry

**Affiliations:** New Albany, Ind.


### PLACEBO IN DENTAL PRACTICE

BY J. C. HENRY, NEW ALBANY, IND.

It may seem strange to many of our profession that it is our duty occasionally to prescribe what in medical parlance is termed a placebo.

If we correctly diagnose many cases placed in our care, and have any sympathy for suffering humanity, we will often witness the happy result of a placebo.

In that mysterious nervous disease hysteria, the dental organs are sometimes involved.

I was engaged in a case about three months ago, that presents some interesting features to the dental practitioner. I was called upon by a gentlemen to visit his wife, stating that she was suffering intense pain from decayed teeth, and would like me to fill them. Instead of preparing myself for such an operation I took my forceps and accompanied him.

Upon examination I found a well defined case of hysteria. The patient referred me to her teeth. I found them decayed, but not a single nerve exposed, nor any pain in the use of the excavator. It was her desire that I should prepare myself and fill the teeth then and there. I did so, knowing all the while that the pain was referred to the teeth, but the difficulty did not originate there.

I had not progressed far with the operation until I found that her husband through kindness, yet through ignorance, had been medicating her upon morphia and brandy to relieve the pain, but he had failed ; and I am of the opinion that any remedy directly from his hands would have failed.

The operation of filling lasted about five hours, and during that time I was invited by the husband seven times to leave the room that she might urinate—a prominent symptom in this disease. When the operation was completed she expressed herself as feeling entirely free from pain, and in a few hours able to leave her room. The remedy I applied I think was the very best. It served a double purpose, that of preserving the teeth and relieving the imaginary pain.

Had the remedy not been so complete I might have been more rational in the treatment, and added Tinct. Valeria internally ; and I doubt not any intelligent medical friend would have said I was wise had I added a half-dozen bread pills.

---

THE most curious question connected with dental caries is the immunity of animals therefrom. Especially are those that gnaw, free from those troubles. It is said that there are South Sea Islanders with remarkably fine teeth, who file them off occasionally. The latter needs confirmation. At all events it is evident that rapid decay takes place on a surface protected from wear by deposit of "tartar," or by the constant presence of other foreign material. There is much yet to learn of enamel. F.

